# Pulmonary arterial remodelling by deficiency of peroxisome proliferator-activated receptor-γ in murine vascular smooth muscle cells occurs independently of obesity-related pulmonary hypertension

**DOI:** 10.1186/s12931-019-1003-4

**Published:** 2019-02-27

**Authors:** Evren Caglayan, Manuela Trappiel, Arnica Behringer, Eva Maria Berghausen, Margarete Odenthal, Ernst Wellnhofer, Kai Kappert

**Affiliations:** 1 Klinik III für Innere Medizin, University of Cologne Heart Center, Cologne, Germany; 2 Center for Molecular Medine Cologne (CMMC), Cologne Cardiovascular Research Center (CCRC), University of Cologne, Cologne, Germany; 3 Berlin Institute of Health, Institute of Laboratory Medicine, Clinical Chemistry and Pathobiochemistry, Center for Cardiovascular Research (CCR), Charité – Universitätsmedizin Berlin, corporate member of Freie Universität Berlin, Humboldt-Universität zu Berlin, Berlin, Germany; 4 Department of Pathology, University of Cologne, Cologne, Germany; 5 Department of Cardiology, German Heart Center Berlin, Berlin, Germany; 6 Department of Cardiology, University Medicine Rostock, Rostock, Germany; 7 DZHK (German Centre for Cardiovascular Research), partner site Berlin, Berlin, Germany

**Keywords:** Pulmonary hypertension, VSMC, PPARgamma, Insulin resistance, Obesity

## Abstract

**Background:**

Obesity is associated with cardiovascular complications, including pulmonary hypertension (PH). Reports suggest that peroxisome proliferator-activated receptor-γ (PPARγ) has direct action in preventing vascular remodelling in PH. Here we dissected the specific role of high-fat-diet (HFD)-induced obesity and vascular smooth muscle cell (VSMC)-PPARγ for remodelling of small pulmonary arteries.

**Methods:**

Wild-type (WT) and VSMC-specific PPARγ-knockout (Sm*Pparγ*^−/−^) mice were fed a low-fat-diet (LFD, 10% kcal from fat) or HFD (60% kcal from fat) for 24 weeks. Mice were metabolically phenotyped (e.g. weight development, insulin/glucose tolerance) at the beginning, and after 12 and 24 weeks, respectively. At 24 weeks additionally pulmonary pressure, heart structure, pulmonary vascular muscularization together with gene and protein expression in heart and lung tissues were determined.

**Results:**

HFD increased right ventricular systolic pressure (RVSP) to a similar extent in WT and Sm*Pparγ*^−/−^ mice. HFD decreased glucose tolerance and insulin sensitivity in both WT and Sm*Pparγ*^−/−^ mice. Importantly, the increase in RVSP correlated with the degree of insulin resistance. However, VSMC-PPARγ deficiency increased pulmonary vascular muscularization independently of the diet-induced rise in RVSP. This increase was associated with elevated expression of early growth response protein 1 in heart and osteopontin in lung tissue.

**Conclusions:**

Here we demonstrate a correlation of insulin resistance and pulmonary pressure. Further, deficiency of PPARγ in VSMCs diet-independently leads to increased pulmonary vascular muscularization.

## Background

Pulmonary arterial hypertension (PAH) is a disease of the small pulmonary arteries that involves vascular proliferation, remodelling, vasoconstriction and thrombosis [1, 2]. These changes lead to a progressive increase in pulmonary vascular resistance, which results in elevated pulmonary artery pressure of ≥25 mmHg, right ventricular failure and premature death in humans [3, 4]. Cells within the vessel wall, including vascular smooth muscle cells (VSMCs), trigger, sustain and regulate these remodelling processes [5]. PAH occurs genetically [6–8], idiopathically or secondary after e.g. heart failure, chronic lung disease, pulmonary embolism [3]. To date, no curing approach has been established, thus, patients are treated symptomatically to improve their quality of life [3].

Clinical and experimental observations suggest a connection between obesity, insulin resistance and VSMC-driven vascular remodelling including PAH [9, 10], while the molecular mechanisms remain elusive. Patients characterized with PAH were shown to be more overweight [11]. Obesity also triggers comorbidities such as obstructive sleep apnea (OSA) and obesity hypoventilation syndrome (OHS) in patients, directly or indirectly impacting PAH. Indeed, the prevalence of PAH in patients with obesity has been reported ranging from 20 to 47% (body-mass-index of 32 kg/m^2^ and 37 kg/m^2^, respectively) [11–13]. Whether diabetes contributes to the risk for PAH or vice versa is a matter of debate. Nonetheless, compared to the overall population, patients with insulin resistance or diabetes have a doubled risk to develop PAH [9, 10]. Further, patients with PAH and diabetes have a significantly reduced walking capacity (six-minutes walking distance), reduced survival and increased risk for pulmonary venous disease with hemodynamics suggesting higher right ventricular diastolic dysfunction. Moreover, diabetics are characterized by significantly higher prevalence of pulmonary embolism [14].

In lung tissue of patients with PAH the expression of peroxisome proliferator-activated receptor-γ (PPARγ) is reduced [15–17]. Moreover, PPARγ agonists have been established in anti-diabetic treatment with beneficial impact on vascular remodelling [18, 19].

Pharmacological activation of PPARγ in VSMCs was shown to decrease their proliferation and migration [20]. Furthermore, in vitro experiments demonstrated that insulin is able to maintain VSMCs quiescence and counteracting growth factor induced dedifferentiation via the phosphoinositide 3-kinase (PI3K) pathway [21]. On the other hand, insulin promotes VSMC migration. During insulin resistance, PI3K signaling is impaired, which may impact on pulmonary vascular remodelling and de novo muscularization of non-muscularized small vessels. While activation of PPARγ restores insulin signaling, the impact of PPARγ in VSMCs in obesity-related pulmonary remodelling has not been determined in detail.

The purpose of the study was to address the role of PPARγ in VSMCs in a model of high fat-diet (HFD) induced obesity. Therefore, we evaluated the impact of HFD on right ventricular systolic pressure (RVSP), pulmonary muscularization and metabolic changes in WT and Sm*Pparγ*^−/−^ mice.

## Methods

### Animals

Sm*Pparγ*^−/−^ mice were generated using transgenic mice expressing Cre-recombinase under the control of the smooth muscle protein 22-alpha promotor (The Jackson Laboratory, Bar Harbor, Maine) and homozygously floxed PPARγ mice [22]. Eight to 12 week old littermate male WT (Cre-) and Sm*Pparγ*^−/−^ (Cre+) mice on a mixed genetic background (C57BL/6, SJL, DBA/2J, C3H) were randomized as follows: WT and Sm*Pparγ*^−/−^ fed ad libitum a low-fat diet (LFD) (10% kcal from fat; Brogaarden, Gentofte, Denmark; D12450J) and WT and Sm*Pparγ*^−/−^ fed ad libitum a high-fat diet (HFD) (60% kcal from fat; Brogaarden; D12492) for 24 weeks. Mice were housed at room temperature with a 12 h light/dark cycle. After sacrificing, organs were excised, weighed, shock-frozen in liquid nitrogen and stored at − 80 °C until further investigation.

### Metabolic phenotyping (body weight, food intake, glucose tolerance test, insulin tolerance test), right ventricular systolic pressure and systemic blood pressure

Body weight was measured twice weekly throughout the study. Food and water intake were measured for up to 24 h in a LabMaster (TSE Systems; Bad Homburg, Germany) after 12 weeks and at the end of the experiment (24 weeks). At the beginning and after 12 and 24 weeks, intraperitoneal (i.p.) insulin tolerance tests (ITT) using a dose of 0.5 U/kg insulin (Insuman® Rapid, Sanofi Aventis, Berlin, Germany) and i.p. glucose tolerance tests (GTT) with 1 g/kg glucose (Glucosteril, Fresenius, Bad Homburg, Germany) were carried out in ~ 4 h and ~ 12 h fasted mice, respectively. Right ventricular systolic pressure (RVSP), a measure for pulmonary arterial systolic pressure (PASP), was recorded with a Millar® microtip catheter (SPR1000), which was inserted into the right ventricle (RV) through the jugular vein. Systemic arterial pressure (SAP) was measured in the carotid artery. The catheter signal was amplified using a PowerLab® amplifier and converted to corresponding pressure curves using LabChart7® software (AD Instruments, Sydney, Australia). To determine the individual SAP and RVSP three to five tracings at different time points were used.

### Lung tissue preparation and morphometric analysis

Lungs were perfused with ice cold phosphate buffered saline (PBS) and subsequently with 1% paraformaldehyde (PFA), extracted and fixed in 4% PFA. After dehydration, lungs were embedded in paraffin using standard procedures. To quantify the degree of muscularization of pulmonary arteries, 3 μm tissue sections were stained for α-smooth muscle actin (Sigma-Aldrich, #2547) and von Willebrand factor (Dako, A0082) using standard immunohistochemical protocols. The degree of muscularization was defined by α-smooth muscle actin positive parts as percentage of the total pulmonary artery cross section: non-muscularized: < 25%, muscularized: ≥ 25%. Data are shown as ratio of muscularized to non-muscularized arteries. Furthermore, the medial wall thickness was determined in vessels with a diameter of 20–70 μm as well as the lumen area (defined as the area within the lamina elastic interna). Osteopontin was visualized by standard immunohistochemistry using anti-osteopontin antibodies (polyclonal anti-osteopontin, #AF808, R&D Systems) and immunoreactive area was quantified in five animals per group.

### Isolation of primary pulmonary arterial vascular smooth muscle cells

The pulmonary artery was dissected and perfused with ice-cold PBS with 1% penicillin/streptomycin (P/S). The artery was incubated in an enzyme mixture (collagenase type I, elastase, trypsin inhibitor) in DMEM supplemented with 20% fetal bovine serum (FBS) for 15 min at 37 °C. To terminate the digestion procedure the arteries were washed with PBS with P/S. The surrounding adventitia and endothelium was mechanically removed, and the artery was cut into pieces and incubated in the enzyme mixture for additional 90 min at 37 °C. Afterwards, primary PASMCs were extracted by centrifugation and transferred to cell culture plates. PASMCs were cultured in DMEM containing 20% FBS and P/S at 37 °C and 5% CO_2_.

### Immunoblotting

Standard immunoblotting was performed using PAGE after protein isolation from aortae. The following primary antibodies were used for protein expression analyses: monoclonal anti-PPARγ (#2443, Cell Signaling), anti-GAPDH (#MAB374, Millipore).

### Immunofluorescence

Isolated PASMCs were incubated on glass tissue slides and fixed with 4% PFA. Slides were washed three times in ice-cold PBS prior to permeabilization with Triton-X. Following one additional washing step, cells were blocked with 5% BSA and incubated with antibodies against α-smooth muscle actin (#2547, Sigma Aldrich) and PPARγ (#2443, Cell Signaling). Slides were washed, and incubated with secondary antibodies (anti-rabbit Alexa Fluor 488 and Cy3-conjugated anti-rabbit IgG, Jackson Immuno Research) and counterstained with 4′,6-diamidino-2-phenylindole (DAPI) for visualization of cell nuclei.

### Assessment of right ventricular hypertrophy

After post mortem isolation of the heart, the right ventricle (RV) was separated from the left ventricle (LV) and ventricular septum (S), and the wet weights were determined. RV hypertrophy was calculated using the Fulton’s index: RV/(LV + S) [23]. Additionally, 3 μm paraffin sections of the RV and LV were stained with hematoxylin and eosin. Cardiomyocyte cross-sectional area (CSA) was determined in the RV and LV as measures of cardiac remodelling. An area of ~ 50 cardiomyocytes in each ventricle from *n* = 4–5 animals per group were evaluated. Microscopic images were analyzed in an observer-blinded manner applying Cell D Imaging Software.

### Gene expression profiling (quantitative real-time PCR)

RNA was isolated utilizing the RNeasy Mini Kit (Qiagen, Hilden, Germany) according to the manufacturer’s instruction for purification from cells and heart tissue. Synthesis of cDNA was performed using the High Capacity RNA-to-cDNA™ Kit (Applied Biosystems, Darmstadt, Germany). Gene expression analysis by quantitative real-time polymerase chain reaction (qPCR) (SybrGreen, Applied Biosystems) was performed in duplicate with an Mx3000P cycler (Stratagene; Agilent Technologies) and normalized to the housekeeping gene 18S. The following primer sequences (at final concentrations of 100 nM) were used: 18S rRNA (*Rn18s*) 5′-GGACTCTTTCGAGGCCCTGTA-3′, 5′-CACCAGACTTGCCCTCCAAT-3′; Osteopontin (*SPP1*) 5′-CTCCAATCGTCCCTACAGTCG-3′, 5′-AGGTCCTCATCTGTGGCATC-3′, Egr-1 (*EGR1*) 5′-CCGAGCGAACAACCCTATGA-3′, 5′-CGAGTCGTTTGGCTGGGATA-3′; BNP (*NPPB*) 5′-GGTCCAGCAGAGACCTCAAAA-3′, 5′-GCCAGGAGGTCTTCCTACAAC-3′ (forward and reverse, respectively).

### Statistical analysis

Data were expressed as means ± SEM or medians and ranges in case of data without normal distribution. We analyzed a full two-factor linear model with repeated measurements for the glucose tolerance test (GTT) and the insulin tolerance test (ITT) with SPSS V25 (IBM). The model was constant + diet + genotype + diet * genotype with measurement time series GTT/ITT as inner subject factor. Difference to baseline was chosen as within-subject contrast. The assumption of normal data distribution was checked for 2 × 2 subgroups by a Kolmogorov-Smirnov test with Lilliefors correction. We performed a Box-test to check for homogeneity of covariances of different groups. The homogeneity assumption did not hold, but Pillai-Bartlett trace was found to be more robust than alternatives when the assumption of homogeneity was violated but sample sizes of the groups were equal [24]. If the maximum ratio of sample sizes was 1.5:1 or less, the F statistic is reported to be robust against violation of the homogeneity of variances assumption [25]. This condition holds since our subgroup sample sizes were nearly equal. We report significance of multivariate analysis based on exact F-statistics and Pillai trace. For further testing the with-in subject effects we tested for sphericity by a Mauchly test and used an appropriate correction (e.g. Huynh Feldt) for the within-subject effects. Homogeneity of variance between within-subject contrasts was asserted by a Levene test in GTT and ITT. Significance of contrasts was assessed by an F-test. Model effects (main effects and interactions) were compared with Tukey correction. A full model univariate two-factor ANOVA was performed with other parameters to test for factor (diet, genotype) effects and interaction between factors. Tests for heteroskedasticity were considered. In case of deviation from normal distribution a logarithmic transformation of the parameter was performed. Transformed parameters did not significantly deviate from normal distribution. Additionally, non-parametric tests with original parameters (Mann-Whitney U, Kruskal Wallis) were performed. *P*-values are given as numbers with the exception of very small *p*-values (*p* < 0.001). Alpha< 0.05 was regarded as significant. P-values near alpha are referred to as borderline. Due to the small power type 2 errors are a concern and borderline significance may be neither an assertion nor a rejection of the null-hypothesis. Trends were assessed by correlation analysis.

## Results

### Impact of Sm*Pparγ*^−/−^ on body weight

**Fig. 1:**
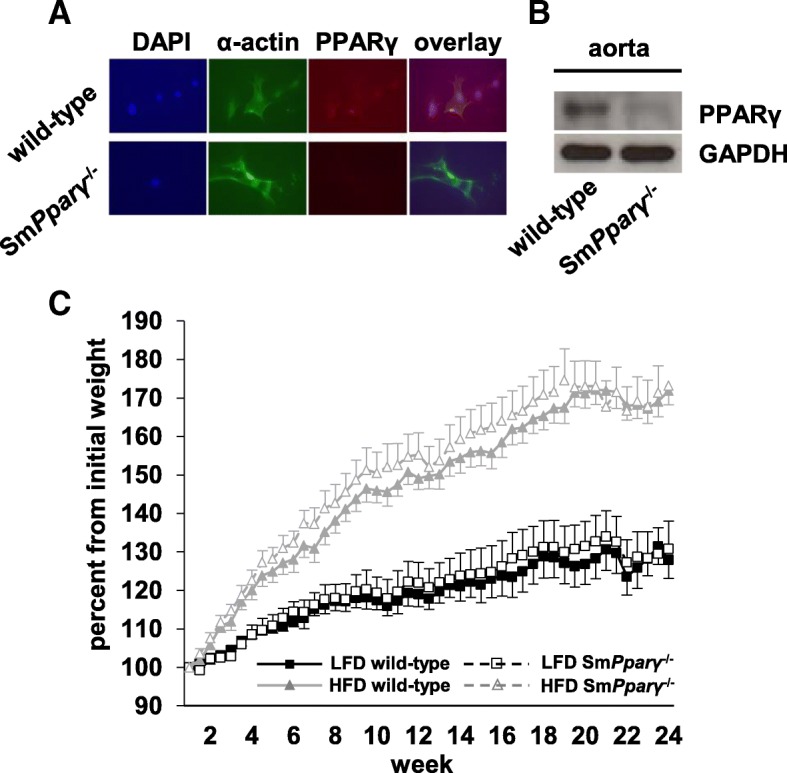
**a** Immunofluorescent stainings (magnification × 400) of isolated PASMCs derived from WT and Sm*Pparγ*^−/−^ mice. Cells from Sm*Pparγ*^−/−^ mice displayed lack of PPARγ immunoreactivity. **b** Immunoblotting analyses of isolated aortae from WT and Sm*Pparγ*^−/−^ mice also demonstrates marked reduction in PPARγ expression in vasculature from Sm*Pparγ*^−/−^ animals. **c** Weight gain curve of WT and Sm*Pparγ*^−/−^ mice on low fat diet (LFD) or high fat diet (HFD). Significant differences (*p* < 0.05) were detectable between LFD- and HFD-fed mice, regardless of the genotype, starting after 2 weeks of feeding (*n* = 10–17 per group). For better visualization of weight curves, significances between LFD and HFD of the same genotypes are not depicted

As a prerequisite for evaluating the relevance of PPARγ in VSMCs in PAH, we analyzed expression levels in isolated PASMCs from WT and Sm*Pparγ*^−/−^ mice. Immunofluorescence of PASMCs revealed that expression of PPARγ was virtually lost in tissues derived from Sm*Pparγ*^−/−^, whereas clearly detectable in WT mice (Fig. 1a). The VSMC-specific knockout of PPARγ was further validated in isolated aortae using immunoblotting procedures (Fig. 1b). Thus, Sm*Pparγ*^−/−^ mice served as a valid animal model for analyses of the impact of PPARγ in VSMCs for metabolic disturbances and pulmonary vascular changes.

WT and Sm*Pparγ*^−/−^ mice were subjected to either a low fat-diet (LFD) or a high fat-diet (HFD) for a total of 24 weeks. Both LFD- (~ 25% of body weight increase) and HFD-fed (~ 70% of body weight increase) mice displayed significant weight gain over time (Fig. 1c). Loss of PPARγ in VSMC (Sm*Pparγ*^−/−^) in HFD mice further enhanced body weight.

### Metabolic characterization of Sm*Pparγ*^−/−^ mice

**Fig. 2:**
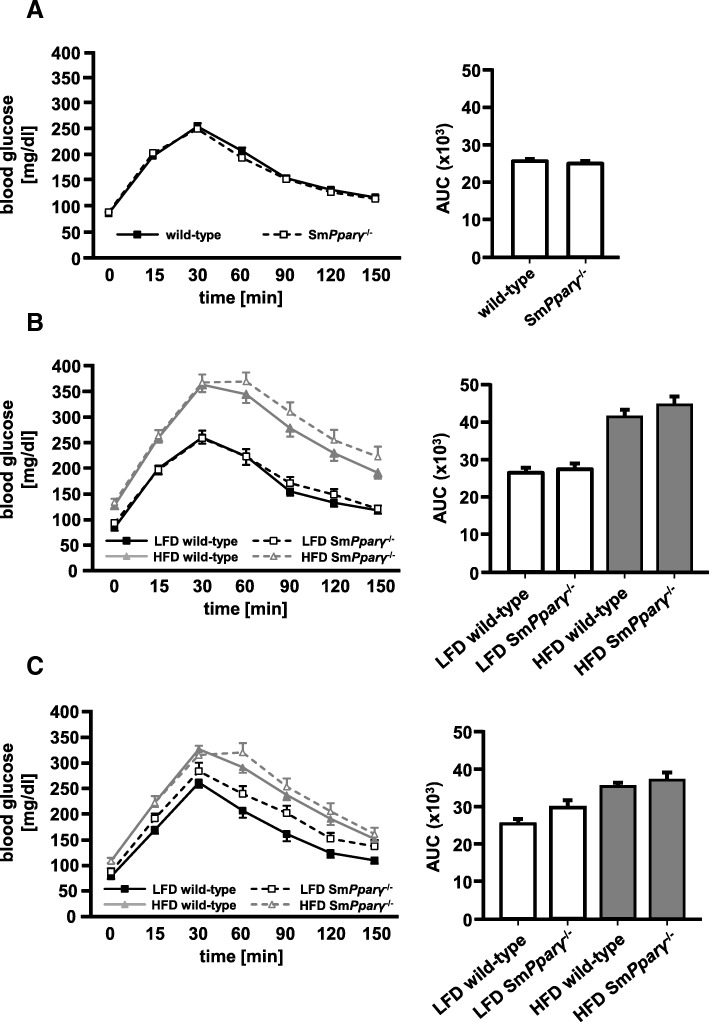
Glucose tolerance tests (GTT) were conducted after fasting **a**) at week 0, **b**) after 12 weeks of LFD and HFD, and **c**) after 24 weeks of LFD and HFD. The corresponding AUC (*right*) was calculated (*n* = 43–44) (**a**), n = 10–12 for LFD and *n* = 30–32 for HFD (**b**), n = 10–17 mice per genotype (WT and Sm*Pparγ*^−/−^) and diet (**c**). For better visualization of glucose curves, significances (*p* < 0.05) between LFD and HFD of the same genotypes are not depicted. A linear two factor repeated measurement model with interaction was performed. The intervention (GTT at 12 weeks) as within-subject factor was highly significant (*p* < 0.001). The interaction of GTT at 12 weeks with diet was significant with p < 0.001, whereas the interaction of GTT with genotype was not significant (*p* = 0.262). There was no three-way interaction of the GTT challenge with diet and genotype (*p* = 0.780). For further testing the with-in subject effects at 12 weeks testing for sphericity was performed by a Mauchly test (*p* = 0.057). The Huynh Feldt correction was used for the within-subject effects. The intervention (GTT at 24 weeks) as within-subject factor was significant (*p* < 0.001). The interaction of GTT at 24 weeks with diet was significant with *p* = 0.003, whereas the interaction GTT with genotype was not significant (*p* = 0.284). The three-way interaction of GTT with diet and genotype was not significant (*p* = 0.859). Homogeneity of variance between within-subject contrasts was asserted by a Levene test in GTT at 24 weeks. Significance of contrasts was assessed by an F-test. In GTT at 24 weeks the change of contrast was significant at all measurement time points (*p* < 0.001). Diet had a significant impact on measurements at 60, 90 and 120 min (*p* < 0.001, *p* = 0.009, *p* = 0.011). Genotype had a possible impact on the measurement at 60 min (*p* = 0.066) in GTT. There was no significant interaction of diet with genotype (*p* = 0.887)

All study groups were subjected to repetitive metabolic phenotyping. Blood glucose levels at baseline GTT (0 weeks) displayed a similar curve pattern in WT and Sm*Pparγ*^−/−^ mice, without evidence of a genotype-specific difference (Fig. 2a). After 12 weeks of diet (Fig. 2b), HFD-fed mice displayed significantly higher fasting glucose levels (*p* < 0.001) and higher serum glucose throughout the GTT until 150 min, also evidenced by quantification of the area under the curve (AUC; *p* < 0.001 LFD vs. HFD). Highest glucose levels were detected at 30 min in HFD-fed WT mice, while the maximum in HFD-fed Sm*Pparγ*^−/−^ mice was seen at 60 min, indicating glucose intolerance in these mice. After 24 weeks of diet (Fig. 2c), again glucose levels after fasting as well as after glucose challenge in both HFD-treated WT and Sm*Pparγ*^−/−^ mice were higher compared to their genotype-related LFD-treated WT and Sm*Pparγ*^−/−^ animals.

**Fig. 3:**
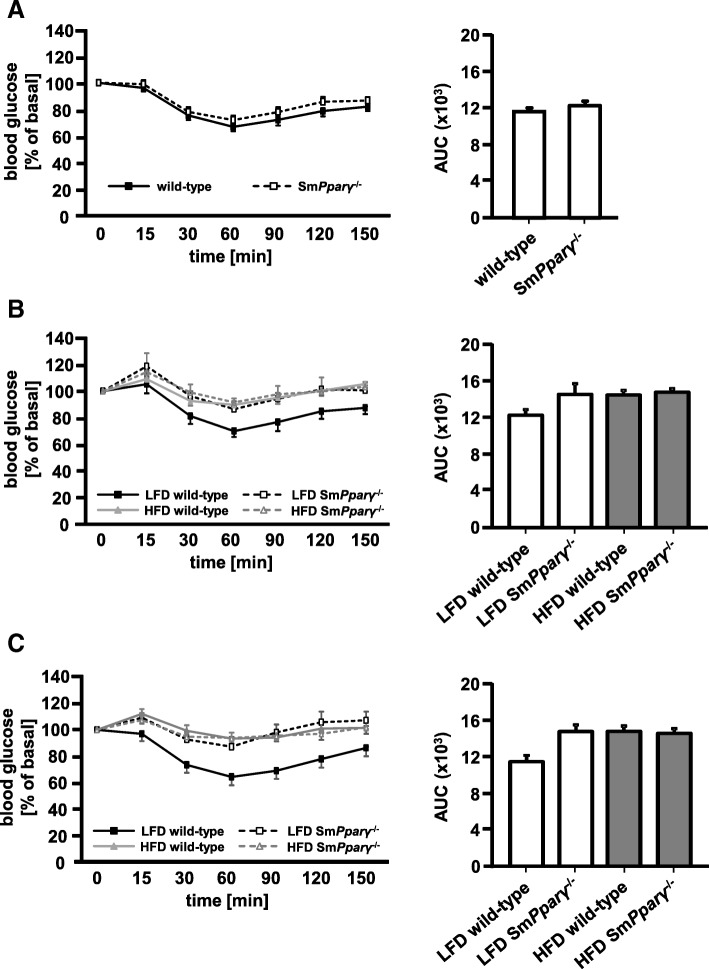
Insulin tolerance tests (ITT) were conducted after fasting **a**) at week 0, **b**) after 12 weeks of LFD and HFD, and **c**) after 24 weeks of LFD and HFD. The corresponding AUC (*right*) was calculated (*n* = 43) (**a**), *n* = 10–12 for LFD and *n* = 31–32 for HFD (**b**), *n* = 10–17 per genotype (WT and Sm*Pparγ*^−/−^) and diet (**c**). Values were normalized to baseline, fasting glucose levels, which were arbitrarily set as 100%. For better visualization of glucose curves, significances (*p* < 0.05) between LFD and HFD of the same genotypes are not depicted. A linear two factor repeated measurement model with interaction was performed. The intervention (ITT at 12 weeks) as within-subject factor was significant (*p* < 0.001). The interaction of ITT at 12 weeks with diet was significant with *p* = 0.043, whereas the interaction with genotype achieved a *p* = 0.072/*p* = 0.94. The three-way interaction of the ITT challenge with diet and genotype was borderline with p = 0.057/*p* = 0.061. For further testing the with-in subject effects at 12 weeks testing for sphericity was performed by a Mauchly test (*p* = 0.039). The Huynh Feldt correction was used for the within-subject effects. The intervention (ITT at 24 weeks) as within-subject factor was significant (*p* < 0.001). The interaction of ITT at 24 weeks with diet was significant with *p* = 0.025, whereas the interaction with genotype achieved a *p* = 0.067. The three-way interaction of ITT with diet and genotype was borderline in this test (*p* = 0.079). Homogeneity of variance between within-subject contrasts was asserted by a Levene test in ITT at 24 weeks. Significance of contrasts was assessed by an F-test. In ITT at 24 weeks the change of contrast was significant at 15, 30, 60 and 90 min (*p* = 0.003, *p* < 0.001, *p* < 0.001, *p* = 0.045). Diet has a significant impact on measurements at 30, 60 and 90 min (*p* = 0.03, *p* = 0.008). There was a significant interaction of diet with genotype in ITT at 15, 30, 60, 90 and 120 min (*p* = 0.042, *p* = 0.028, *p* = 0.035, *p* = 0.015, *p* = 0.014)

At baseline (0 weeks) Sm*Pparγ*^−/−^ displayed no major difference in insulin tolerance compared to their WT littermates (Fig. 3a). After 12 weeks (Fig. 3b) and 24 weeks (Fig. 3c) only LFD-fed WT mice showed preserved insulin sensitivity, while the other groups displayed insulin resistance. Indeed, this pattern became more evident at 24 weeks, now showing a more profound difference of insulin sensitivity in Sm*Pparγ*^−/−^ LFD-fed mice compared to their WT littermates. Thus, Sm*Pparγ*^−/−^ in LFD resembled the impact of HFD in WT mice. This underlines an impact of smooth muscle PPARγ for whole body insulin sensitivity. Noteworthy, HFD did not further significantly increase the level of insulin resistance in Sm*Pparγ*^−/−^ (Fig. 3c).

We conclude that both diet and genotype have an effect on glucose metabolism with a relevant interaction.

### Food intake and organ weights

Food intake was equal between LFD wild-type and LFD Sm*Pparγ*^−/−^ mice (1.80 ± 0.34 g/18 h and 1.75 ± 0.38 g/18 h) and between HFD wild-type and HFD Sm*Pparγ*^−/−^ mice (1.42 ± 0.27 g/18 h and 1.44 ± 0.34 g/18 h).

Following sacrificing the animals after 24 weeks, organ measurements were performed. As shown in Table 1, liver and kidney weights were higher in HFD-treated mice compared to LFD-mice. Additionally, perirenal fat and epididymal fat tissue were higher in HFD- vs. LFD-fed mice. In contrast, brown adipose tissue (BAT) remained unchanged under HFD.

**Table 1:** Organ weights in grams (mean ± standard deviation)

	LFD	LFD	HFD	HFD	*P*	n
	wild-type	Sm*Pparγ*^−/−^	wild-type	Sm*Pparγ*^−/−^		
Liver	1.4 ± 0.4	1.6 ± 0.5	2.1 ± 0.9	2.3 ± 0.9	0.579 ^1^	10–16
					0.003 ^2^	
					0.001 ^3^	
					0.221 ^4^	
Kidney	0.5 ± 0.2	0.5 ± 0.2	0.6 ± 0.2	0.6 ± 0.2	0.575 ^1^	10–16
					0.009 ^2^	
					< 0.001 ^3^	
					0.529 ^4^	
AT perirenal	0.5 ± 0.7	0.9 ± 0.4	1.7 ± 1.1	1.7 ± 0.6	0.200 ^1^	10–15
					< 0.001 ^2^	
					0.005 ^3^	
					0.916 ^4^	
BAT	0.2 ± 0.1	0.3 ± 0.1	0.3 ± 0.1	0.3 ± 0.1	n.s.	9–11
AT epididymal	0.8 ± 0.3	1.3 ± 0.3	1.9 ± 0.2	1.7 ± 0.1	0.271 ^1^	9–16
					< 0.001 ^2^	
					0.140 ^3^	
					0.196 ^4^	

Abbreviations: *LFD* low fat-diet, *HFD* high fat-diet, *AT* adipose tissue, *BAT* brown adipose tissue. *n.s.* non-significant; ^1^: LFD wild-type vs. LFD Sm*Pparγ*^−/−^; ^2^: LFD wild-type vs. HFD wild-type; ^3^: LFD Sm*Pparγ*^−/−^ vs. HFD Sm*Pparγ*^−/−^; ^4^: HFD wild-type vs. HFD Sm*Pparγ*^−/−^

### Hemodynamic characterization in LFD- and HFD- fed WT and KO mice

**Fig. 4:**
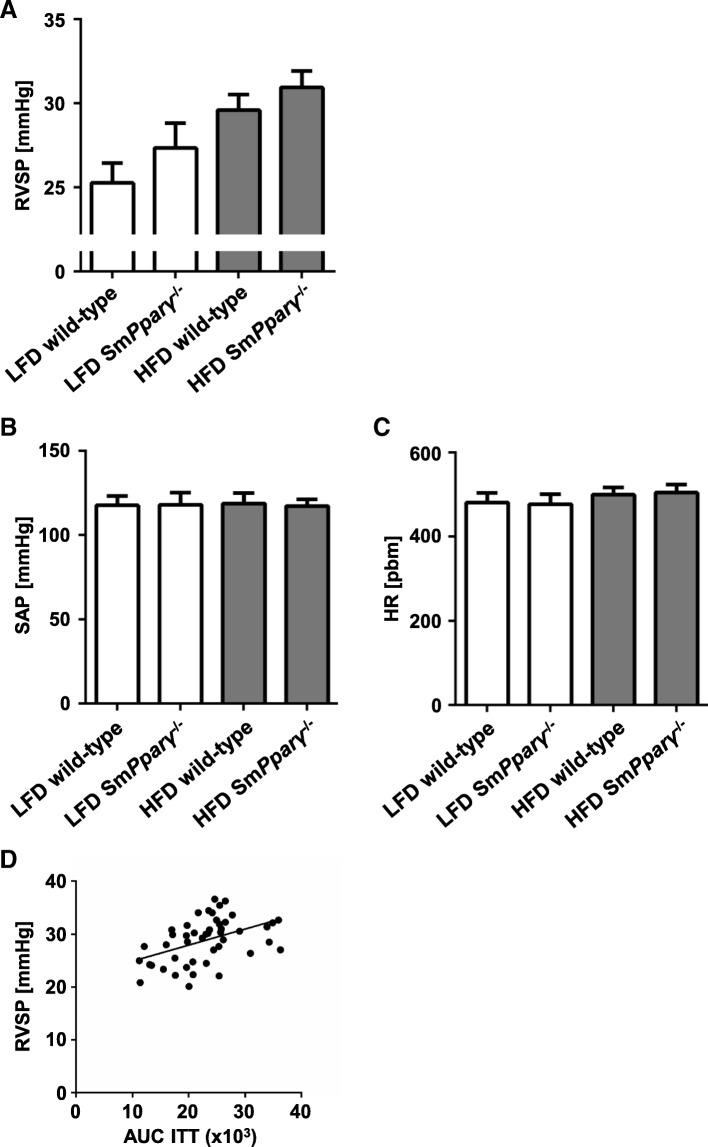
**a** Right ventricular systolic pressure (RVSP) was determined as stated in the Materials and Methods section in mice after 24 weeks in WT and Sm*Pparγ*^−/−^ mice subjected to LFD or HFD. In a two-factor ANOVA with interaction the diet had the predominant impact with *p* = 0.01. The effect of genotype did not reach significance (*p* = 0.143), and there was no interaction (*p* = 0.754). **b** Systemic arterial pressure (SAP, mmHg) in mice after 24 weeks in WT and Sm*Pparγ*^−/−^ mice subjected to LFD or HFD is shown. **c** Heart rate (beats per minute, bpm) in mice after 24 weeks in WT and Sm*Pparγ*^−/−^ mice subjected to LFD or HFD (*n* = 8–15 per group). **d** Analyses of correlation between right ventricular systolic pressure (RVSP) and area under the curve (AUC) of absolute glucose levels in ITT determined at the 24 week time point for all analyzed animal groups. Pearson r = 0.45, *p* < 0.01

**Fig. 5:**
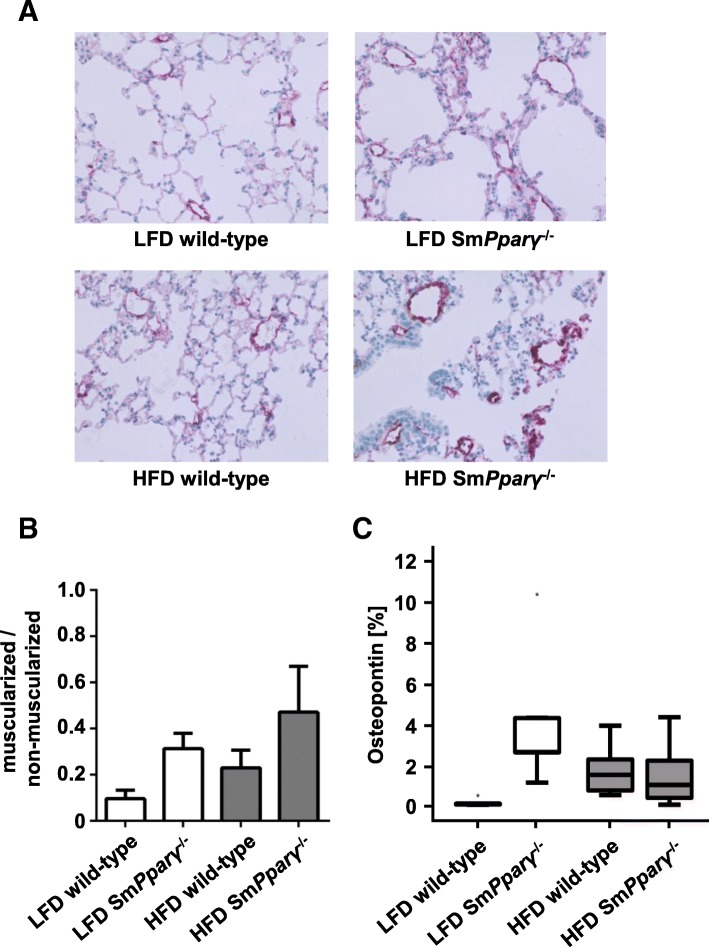
Effect of genotype and diet on pulmonary vascular remodelling (arteries sized 20–70 μm) in monitored study groups: **a** Representative pictures from immunohistochemical stainings of pulmonary tissues in all analyzed animal groups using α-smooth muscle actin antibodies for visualization of vascular muscularization. **b** Quantification of vascular muscularization was performed in lung arteries. Shown are the ratios of non- and muscularized pulmonary arteries (*n* = 23–27 arteries / *N* = 5 animals). There was a borderline effect (*p* = 0.06) of genotype without interaction with diet (a two-factor linear model with interaction and a non-parametric Kruskal-Wallis test was performed). **c** Shown are quantifications of lung immunohistochemical stainings with antibodies against osteopontin for detection of extracellular matrix remodelling in each study group (*n* = 5). A univariate general two factor model with interactions was used for testing. A logarithmic transformation was used to avoid violation of homodekasticity. Additional non-parametric tests (Kruskal-Wallis, Jonckheere-Terpstra) were performed. The *p*-values were significant for genotype (*p* < 0.001) and diet (*p* = 0.021) with significant interaction of genotype with diet (*p* = 0.001)

**Fig. 6:**
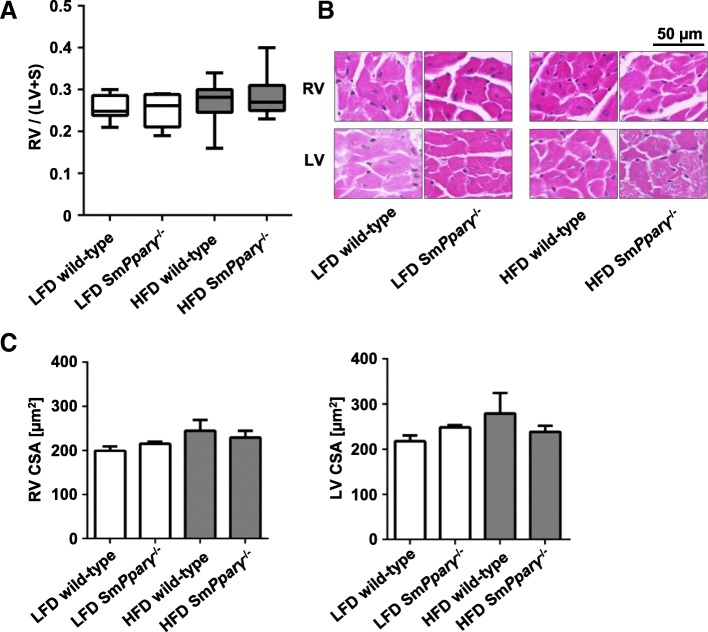
Impact of genotype (WT and Sm*Pparγ*^−/−^) and diet on the right (RV) and left ventricle (LV) remodelling and hypertrophy: **a** RV to LV + septum (S) ratios (= Fulton’s Index) from mice of each study group are shown at the 24 week time point (*n* = 8–15 per group). **b** Shown are representative hematoxylin-eosin stainings of the RV and the LV derived from all analyzed animal groups (scale bar = 50 μm). Large pictures were digitalized at × 40, insets are shown for better visualization. **c** Quantifications of the myocyte cross section area (in μm^2^) in the RV and LV in each study group (*n* = 16–50 myocytes / *N* = 4–5 animals). For **a**, **c** and **d** a two-factor linear model with interaction and a non-parametric Kruskal-Wallis test was performed

Right ventricular systolic pressure (RVSP), as a measure for pulmonary artery systolic pressure (PASP), was determined, along with systemic artery pressure (SAP) and heart rate. RVSP was significantly enhanced by HFD without interaction of genotype (Fig. 4a). Of note, SAP and heart rate were comparable in all investigated groups (Figs. 4b, 5 and 6c).

We performed additional statistical analyses for potential correlation between insulin sensitivity and RVSP. As shown in Fig. 4d, a significant correlation was calculated, underlining an association between metabolic disturbances and pulmonary vascular pressure.

### Structural analyses

Muscularization of pulmonary arteries is an early crucial morphological feature of pulmonary vascular remodelling. Sm*Pparγ*^−/−^ enhanced the proportion of muscularized to non-muscularized arteries in both LFD- and HFD-fed animals (Fig. 5a-b), indicating an impact of PPARγ in VSMCs for de novo muscularization of pulmonary arteries.

Sm*Pparγ*^−/−^ LFD-fed mice displayed increased expression of osteopontin (Fig. 5c), an extracellular matrix protein exhibiting both tissue remodelling and inflammatory properties, which further was established as contributing to PAH [26], indicating enhanced extracellular lung tissue remodelling in these mice. Similarly, HFD also enhanced osteopontin content in WT animals.

As a measure of RV hypertrophy, the Fulton’s index was determined post mortem. The Fulton’s index showed a median of 0.26 (95% confidence interval (CI) 0.23–0.28) in LFD WT animals and did not increase in HFD-fed WT mice (median = 0.27, 95% CI 0.24–0.29). There was no marked difference related to the genotype in both diets (Fig. 6a).

Changes in cardiomyocyte morphology are integral components of heart remodelling associated with PAH. Figure 6b depicts representative hematoxylin-eosin stainings and corresponding quantification. We evaluated the cross-sectional area (CSA) of cardiomyocytes as one parameter displaying heart tissue remodelling. Figure 6c depicts that neither HFD nor Sm*Pparγ*^−/−^ reached a significant effect on CSA in the RV and LV after 24 weeks treatment in the subgroups.

**Fig. 7:**
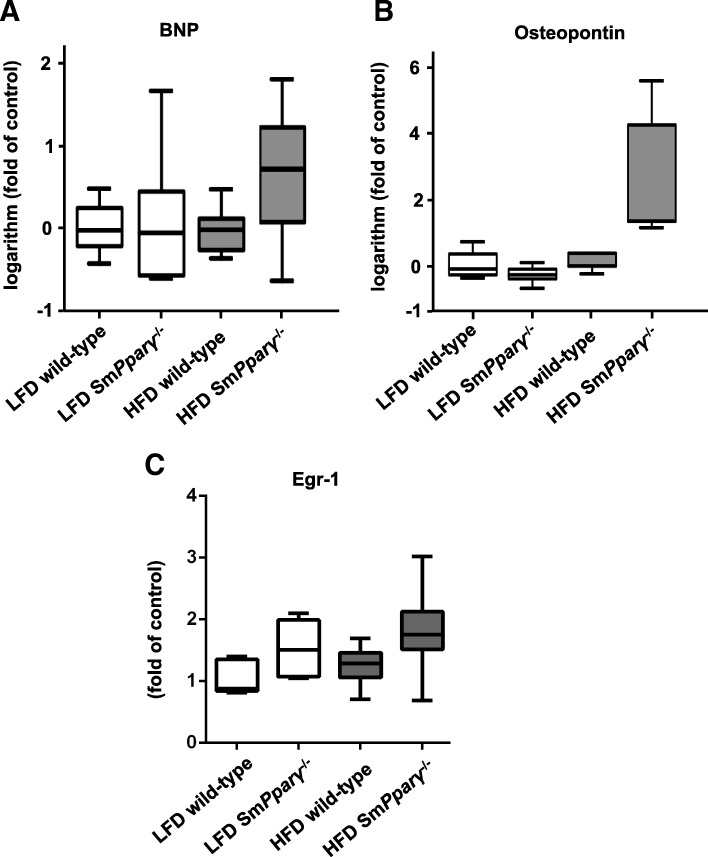
**a** Relative logarithmized gene expression of brain natriuretic peptide (BNP) in the right ventricle (RV) in each study group. Transcript levels were determined by qPCR analysis, and were normalized to the expression of *18S* and to the expression of LFD WT mice (*n* = 6–8 per group). **b** Relative logarithmized gene expression analyses of osteopontin in the RV in each study group. Transcript levels were determined by qPCR analysis, and were normalized to the expression of *18S* and to the expression of LFD WT (*n* = 4–6 per group). By a two-factor linear model with interaction and a non-parametric Kruskal-Wallis test we detected that diet had a significant effect on osteopontin (*p* = 0.01) with borderline interaction with genotype (*p* = 0.06). **c** Relative gene expression analyses of Egr-1 in the RV in each study group. Transcript levels were determined by qPCR analysis, and were normalized to the expression of *18S*. The expression of LFD WT mice was arbitrarily set as 1 (n = 6–7 per group). By a two-factor linear model with interaction and a non-parametric Kruskal-Wallis test we detected that the genotype had a significant effect on Egr-1 (*p* = 0.019)

Transcript analyses were performed to detect potential differences in gene expression that had yet transferred to only minor morphological changes but, in particular, functional changes in the RV as demonstrated by increased RVSP. Brain natriuretic peptide (BNP) is an established and widely used clinical parameter for heart function and failure, and ventricular remodelling. While BNP was shown being a prognostic marker in PAH [27], others ruled out the NT-proBNP response to exercise as a disease severity assessment marker of PAH [28]. No significant upregulation of BNP was detectable in the RV due to the application of different diets (Fig. 7a). Osteopontin had previously been shown being enhanced in the RV in animal models of PH [29]. HFD upregulated osteopontin in the RV (Fig. 7b). Further, early growth response protein 1 (Egr-1), critically involved in vascular remodelling in PAH [30], was higher expressed in the RV in Sm*Pparγ*^−/−^ mice in both LFD- and HFD treated animals (Fig. 7c).

## Discussion

Here we show that high fat-diet (HFD) in mice, given long-term for 24 weeks, increases body weight and right ventricular systolic pressure (RVSP), a measure of pulmonary arterial systolic pressure (PASP), similarly in WT and Sm*Pparγ*^−/−^ mice. Further, glucose tolerance and insulin sensitivity are impaired by HFD in both genotypes. Importantly, insulin resistance correlates with RVSP. Sm*Pparγ*^−/−^ mice display enhanced muscularization of small pulmonary arteries under both diet conditions and also higher osteopontin deposition in the lung. Further, Sm*Pparγ*^−/−^ display elevated Egr-1 gene expression.

Based on clinical and experimental findings, suggesting an association of obesity, insulin resistance and PAH [9, 10], here we applied an HFD-induced obesity mouse model for evaluation of this association. Since PAH patients are characterized by reduced expression of apolipoprotein E (apoE), earlier experimental settings focused on PAH-development on an apoE^−/−^ background. Male apoE^−/−^ mice not only displayed insulin resistance under HFD-feeding, but also developed higher RVSP along with RV hypertrophy and increased muscularization of small pulmonary arteries [31]. Our study, however, shows that *long-term* HFD alone results in enhanced pulmonary pressure even in WT mice, without apoE^−/−^ background, when given for 24 weeks. These findings are underlined by data, showing significantly enhanced mean pulmonary artery pressure in fatty compared to lean Zucker rats [32]. In addition, male C57BL/6j mice given an HFD for 20 weeks showed PH evidenced by increased right ventricular end-systolic pressure [33]. Further, insulin-resistant apoE-deficient (apoE^−/−^) mice on an HFD spontaneously develop PAH [31] and recently HFD-apoE^−/−^ mice treated with a dual ET_A_/ET_B_ receptor antagonist were characterized by beneficial effects on PAH [34]. Moreover, a 20-week HFD in C57BL/6 mice also led to significantly higher RVSP compared to mice subjected to standard chow diet, which underscores our data, even though the increase in RVSP was milder in our study (4.3 mmHg vs. 16.2 mmHg, respectively) [33], probably due to the mixed genetic background of our used mice (C57BL/6, SJL, DBA/2J, C3H) [35–37]. Indeed, a recent publication compared the susceptibility developing PAH induced by HFD in 36 different mouse strains. These analyses demonstrated significant differences with both resistance (e.g. SJL/J, DBA/2J, C3H/HeJ) and susceptibility (e.g. C57BL/6J) of HFD-induced PAH [37].

As expected, HFD time-dependently induced obesity, changes in body composition, and reduced glucose tolerance and insulin sensitivity [38–40]. Clinical observations have shown that diabetics have a higher risk for development and prevalence of PAH [10, 14, 41], with the causal and underlying mechanism yet to be established. Indeed, others failed to establish a clear association between insulin dysregulation and PAH [42]. Nonetheless, insulin resistance in female PAH patients was associated with a lower six-month event-free survival [10]. In our study, we provide evidence for a significant correlation between RVSP and reduced insulin sensitivity. The changes in RVSP and insulin sensitivity under HFD were further accompanied by increased muscularization of small pulmonary arteries. We and others earlier have shown that osteopontin exerts both proinflammatory and chemoattractant properties in remodelling processes, including experimental PAH [22, 43, 44]. In line, we found enhanced osteopontin deposition under HFD treatment in WT and due to PPARγ-deficiency. Of note, Sm*Pparγ*^−/−^ resulted in higher muscularization of small pulmonary arteries also in LFD mice without enhanced RVSP, suggesting an HFD-independent impact of PPARγ-deficiency.

Even though clinical and experimental correlations between glucose dysregulation and PAH exist, it is still a matter of debate whether obesity and metabolic disturbances are a consequence of PAH, representing only a marker of severe pulmonary vascular disorders, or whether a potential pathogenetic relationship with disease initiation and/ or progression exists. While in patients obesity-related PAH was initially considered secondary to hypoventilation and lung hypoxia resulting from increased mechanical overload in excessive fat tissue [45, 46], some observations have questioned the indirect relationship and favor metabolic and/ or inflammatory pathways triggering PAH [10, 32, 47]. Based on previous findings that PPARγ is less expressed in lung tissue of patients with PAH [16] experimental data showed that insulin-sensitizing PPARγ agonists reduce PAH in rodent models [9, 31, 43, 48, 49]. In addition, with TGFβ1 acting pro-proliferative in VSMCs it was recently demonstrated that pioglitazone even reversed PAH in TGFβ1-overexpressing mice [50] and in the SU5416/hypoxia (SuHx) rat model by normalizing epigenetic and transcriptional regulation [51]. We thus analyzed Sm*Pparγ*^−/−^ mice with regard to both RVSP and the metabolic phenotype in an experimental model of obesity. Sm*Pparγ*^−/−^ mice displayed impaired insulin sensitivity in mice even subjected to LFD. While we analyzed mice at a final age of 32–36 weeks, these data are in line with Hansmann et al. [48] showing that younger SMC *PPARγ*^−/−^ mice develop spontaneously PAH on a regular chow diet. Glucose uptake is mainly driven by peripheral tissues, e.g. skeletal muscle. Food intake was not significantly different between both diet and genotypes in our study. *Off-target* effects of Sm*Pparγ*^−/−^ in other muscle tissue, including skeletal muscle, cannot fully be ruled out. Interestingly, knockout of PPARγ in VSMCs enhanced pulmonary vascular remodelling. Therefore, increased muscularization in small pulmonary arteries, even in LFD-fed mice, is in favor of a rather cell-specific knockout.

The expression of Egr-1 has been shown to be increased in pulmonary vessels of PAH patients [30]. Knockout of PPARγ in VSMCs enhanced Egr-1 gene expression in RV in both LFD- and HFD-fed mice, suggesting an indirect effect due to pulmonary vessel remodelling. Gene expression of osteopontin, an extracellular matrix protein exhibiting both tissue remodelling and inflammatory properties, was higher in HFD Sm*Pparγ*^−/−^, which was also shown earlier in an MCT-induced model in rats [43]. Interestingly, while HFD led to enhanced osteopontin content in lung tissue in WT, also knockout of PPARγ in VSMCs in LFD mice was followed by a significant rise, while there was no further increase in HFD-Sm*Pparγ*^−/−^ as compared to LFD-Sm*Pparγ*^−/−^.

An important limitation of our study is that SM22α-Cre deletes also in myeloid cells therefore non-SMC dependent effects of our observations cannot fully be ruled out. Further, others have shown that transgenic mice (expressing dominant-negative mutations in PPARγ) are characterized by both impaired vasodilation of the thoracic aorta and systolic hypertension [52], implicating a relevant impact on larger arteries. In contrast, we focused on the role of PPARγ in VSMCs impacting on PH and thus small arteries. We cannot fully rule out an additional effect on larger arteries. In our animal model, however, a major impact seems less likely since systemic arterial pressure between genotypes did not differ.

## Conclusions

In conclusion, HFD leads to an increase in pulmonary arterial pressure, substantiating the interplay of metabolic disturbances and pulmonary vascular remodelling. This is also evidenced by a correlation between insulin resistance and RVSP. Knockout of PPARγ in VSMCs resulted in attenuated insulin sensitivity and enhanced pulmonary vascular muscularization. PPARγ in VSMCs is supposed to play a critical role in PAH-associated pulmonary remodelling, in addition to obesity-related pulmonary hypertension.
